# Clinical evaluation of automatic whole-heart and coronary-artery segmentation

**DOI:** 10.1186/1532-429X-11-S1-P220

**Published:** 2009-01-28

**Authors:** Marcel Breeuwer, Pierre Ermes, Bernhard Gerber

**Affiliations:** 1 Philips Healthcare, Best, Netherlands; 2 Louvain University, St. Luc Hospital, Brussels, Belgium

**Keywords:** Manual Correction, Deformable Model, Segmentation Quality, Correction Effort, Fair Quality

## Introduction

With Steady-State Free-Precession (SSFP) MRI the heart and surrounding arteries can be imaged. The resulting image data can be used to inspect the patient-specific cardiac anatomy and to quantify aspects such as left-ventricular volume and wall mass. The data can furthermore be used to detect stenosis in the proximal and middle coronary-artery segments [1]. To simplify the visualization, quantification and stenosis detection, we have developed an almost automatic whole-heart and coronary-artery segmentation method.

## Purpose

We describe the clinical evaluation of the performance of our cardiac MR whole-heart and coronary-artery segmentation method.

## Methods

Our segmentation method was first developed for Computed Tomography [2] and thereafter adapted to MR [3]. The method fully automatically segments the ventricles, atria, and part of the aorta and pulmonary vessels by means of a shape-constrained deformable model, which has been constructed from a large set of representative trainings data. Manual corrections of the segmentation are possible by single mouse clicks on the 3D segmentation surface visualization. The points where the coronary-arteries branch off the aorta (ostia) are also automatically detected. The user has to manually indicate the coronary-artery end points in the image data, these arteries are thereafter automatically tracked [4]. Total time needed for automatic segmentation/tracking is less than 2 min per data set (Dell 670 PC, dual-processor 3 GHz, 3 Gbyte memory).

Our method was evaluated by an experienced clinical cardiologist (third author) on whole-heart MR acquisitions from 50 patients with coronary-artery disease or heart failure (Philips Achieva 1.5 T, typically 150 slices, TE = 2.14 ms, TR = 4.27 ms, flip angle = 86 degree, pixel spacing = 0.7 mm, slice distance = 0.8 mm). The cardiologist visually judged the segmentation quality before and after manual correction (categories: poor, fair, average, good, excellent). The time needed to perform segmentation corrections was recorded and the cardiologist judged the required correction effort (categories: none, small, medium, large).

## Results

**Figure 1:**
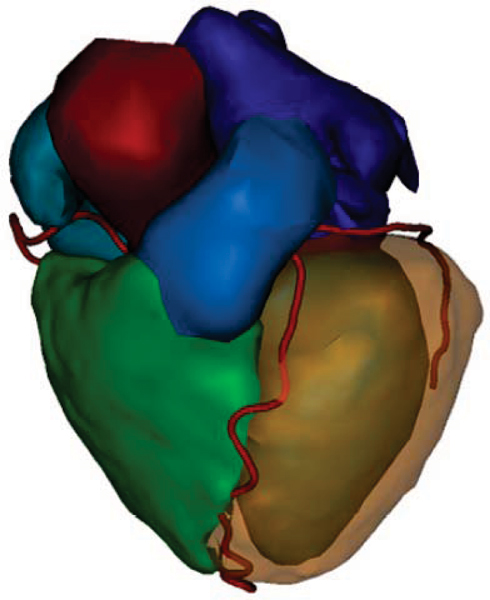
**Example of whole-heart and coronary-artery segmentation (surface rendering with different colour per component)**.

**Figure 2:**
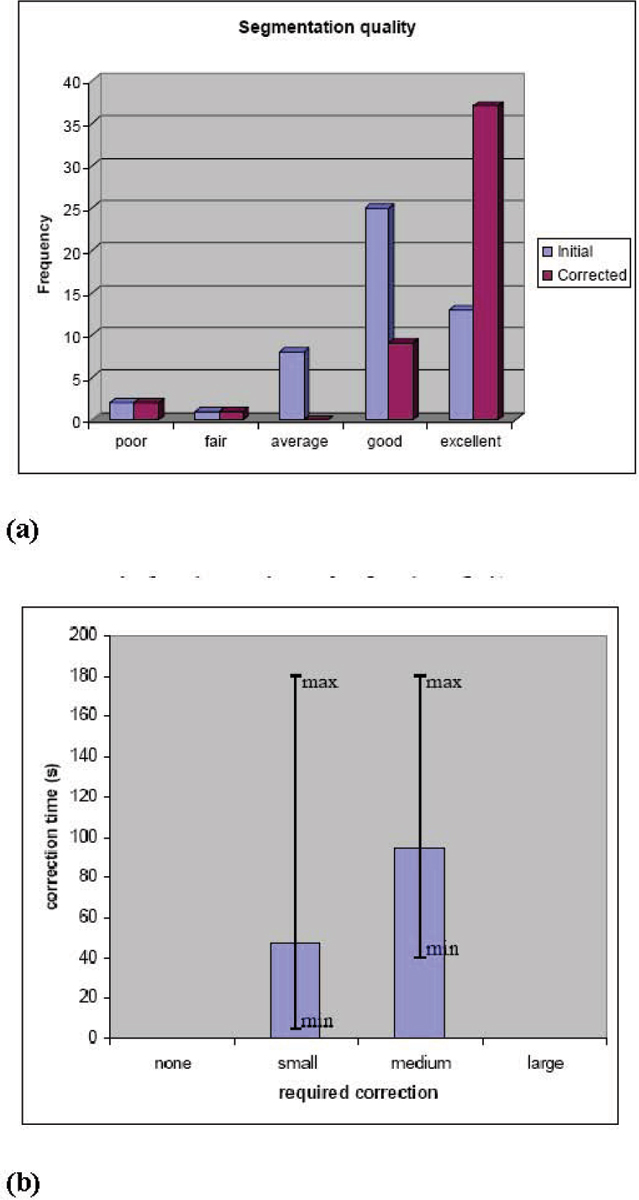
**(a) Histogram of segmentation quality before and after correction (frequency = nr. of cases per quality category)**. **(b)** Average (blue bars), minimum and maximum (lines) correction times, for each of the categories of judged correction effort.

Figure 1 shows an example whole-heart segmentation. Figure 2a shows the histogram of the quality before and after manual correction. Before correction, 47 out of 50 segmentations have an average-to-excellent overall quality. Manual correction significantly improves the quality, after correction 47 out of 50 have a good or excellent quality. Three segmentations have a poor or fair quality, manual correction does not help for these cases. Figure 2b shows the time and judged effort needed to correct segmentations. Only small (average time 45 sec) or medium (average time 94 sec) correction efforts were judged to be needed. The maximum correction time (180 sec) does however not differ for these categories.

## Conclusion

For 47 out of 50 investigated cases (94%), our automatic whole-heart and coronary-artery segmentation method in combination with small or medium correction effort is judged to result in good or excellent segmentation quality.
